# Impact of chemotherapy exposure on tumor mutation burden in advanced colorectal cancer

**DOI:** 10.1038/s41598-026-46050-7

**Published:** 2026-04-06

**Authors:** Tomoya Sudo, Sachiko Nagasu, Toshimitsu Tanaka, Kenji Fujiyoshi, Yusuke Kawamoto, Satoshi Shimamura, Junya Fukuda, Maako Kikuchi, Hirona Shigyo, Kenichi Koushi, Takahiro Shigaki, Naohiro Yoshida, Takefumi Yoshida, Keisuke Miwa, Hironori Koga, Fumihiko Fujita

**Affiliations:** 1 Division of Digestive Surgery, Department of Surgery, Kurume University School of Medicine, Asahi-Machi 67, Kurume, Fukuoka 830-0011 Japan; 2 Research Center for Innovative Cancer Therapy, Kurume University, Kurume, 830-0011 Japan; 3 Division of Gastroenterology, Department of Medicine, Kurume University School of Medicine, Kurume, 830-0011 Japan; 4 Multidisciplinary Treatment Cancer Center, Kurume University, Kurume, 830-0011 Japan

**Keywords:** SNV, CGP, TMB, Colorectal cancer, Chemotherapy exposure, Clinicopathologic factors, Cancer, Computational biology and bioinformatics, Genetics, Oncology

## Abstract

The determinants of tumor mutation burden (TMB) in microsatellite-stable (MSS) colorectal cancer (CRC) remain unclear. This study examined the impact of clinical, environmental, and genomic factors on TMB in advanced CRC using comprehensive genomic profiling (CGP). Eighty-eight patients with advanced or recurrent CRC who underwent CGP were analyzed and compared with 701 MSS CRC cases from a public dataset. Associations between TMB and clinicopathological, environmental, and genomic factors were evaluated. The number of prior chemotherapy regimens was significantly associated with higher TMB. No associations were observed between TMB and alcohol use, smoking history, or specific chemotherapeutic classes. Although variants in *POLE* or *POLD1* were detected, all were non-pathogenic and were not linked to elevated TMB. The top 3 three most frequent genes were consistent with those in the public dataset, although variant frequencies were higher in this cohort. In MSS TMB high CRC, elevated TMB may reflect cumulative treatment-related effects rather than intrinsic hypermutagenic mechanisms, highlighting the need to interpret TMB in the context of therapeutic history.

**Supplementary Information:**

The online version contains supplementary material available at 10.1038/s41598-026-46050-7.

## Introduction

### Current global and Japanese trends in colorectal cancer

Globally, colorectal cancer (CRC) is the fourth most common cancer and the third leading cause of cancer death (https://gco.iarc.who.int/today/en/dataviz/bars?mode=cancer&group_populations=1&types=0_1&sort_by=value1). In Japan, 385,794 people died from cancer in 2022, with CRC being the second leading cause of cancer death after lung cancer (https://www.mhlw.go.jp/toukei/saikin/hw/jinkou/kakutei22/index.html). Consequently, CRC is a critical cancer that needs to be addressed. Standard treatments for CRC include surgery, chemotherapy, radiation therapy, targeted therapy, and immunotherapy, which have all contributed to improved survival rates. However, the 5 year survival rate for stage IV CRC remains below 20% (https://www.cancer.gov/types/colorectal), indicating potential for improvement. Comprehensive genomic profiling (CGP) test is increasingly utilized in clinical practice for patients with advanced or recurrent CRC, helping to identify optimal treatments and clinical trial designs based on CGP results.

#### Key factors in the treatment strategy for advanced CRC

When devising treatment strategies for advanced CRC, the presence of *RAS* mutations and the status of microsatellite instability (MSI)/mismatch repair deficiency (dMMR) or tumor mutation burden (TMB) are extremely important. The presence of *RAS* mutations is associated with resistance to treatments such as cetuximab and panitumumab^1^, limiting selection of regimens for neoadjuvant or adjuvant therapy^2^. In contrast, a high MSI (MSI-high)/dMMR or high TMB (TMB-high) status can indicate suitability for immunotherapy, with substantial resultant therapeutic efficacy.

#### CRC and immunotherapy

The introduction of immune checkpoint inhibitors (ICIs) for the treatment of CRC has contributed to improved therapeutic outcomes. Currently, the criteria for administering ICIs in CRC include MSI-high or dMMR status. However, in the US, only 15–20% of CRC cases exhibit MSI-high/dMMR^3^, and in Japan, this percentage is even lower, at 6–7%. Thus, the prognostic improvements associated with ICIs are currently limited. Meanwhile, a TMB-high status is observed in about 9% of CRC cases, with 3% to 10% of microsatellite stable (MSS) cases being TMB-high^4,5^, underscoring the importance of considering TMB status in the treatment of MSS CRC.

#### Unresolved issues and the purpose of this study

Consideration of TMB status is crucial for determining treatment strategies for many cancer types. It is important to note that TMB is not solely innate and that it can be acquired through treatment and environmental factors. For example, smoking has been identified as an extrinsic factor contributing to increased TMB in lung squamous cell carcinoma, while ultraviolet radiation is a recognized extrinsic contributor to elevated TMB in cutaneous melanoma^6^. In CRC, environmental toxins, microbiota, and pharmacological agents have been implicated in TMB modulation^7^; however, the extent to which anticancer chemotherapy, as a treatment-related extrinsic factor, influences TMB has not yet been clearly established. On the intrinsic side, CRCs harboring *POLE/POLD1* gene variants exhibit elevated TMB and represent predictive biomarkers for ICI efficacy^5,8,9^, with additional reports implicating other DNA-repair–related gene mutations. In contrast, involvement of other DNA-repair related genes in TMB elevation has not been explicitly demonstrated.

In this study, we analyzed CGP data from CRC cases at our institution to characterize prevalent gene variant profiles. We also investigated the potential impact of clinicopathological and extrinsic environmental factors—including alcohol consumption, smoking, and exposure to chemotherapy—on TMB.

## Materials and methods

### Patients and CGP data

This study included 88 patients at our institution with either stage IV CRC or advanced recurrent CRC post-surgical intervention. During treatment, these patients were deemed suitable for CGP, and either FoundationOne CDx (F1CDx) or FoundationOne Liquid CDx (F1LCDx) tests (Foundation Medicine, Inc, Cambridge, MA, US) were conducted from 2019 to 2025. An expert panel (EP) at Kurume University Hospital reviewed the results, and the EP final report data were used. Additionally, data extraction was performed from extensible markup language (XML) files obtained from the F1CDx and F1LCDx test raw data. These XML files were then converted into the data file formats required for subsequent analyses (Supplementary Table 1, 2, and 3).

For the reference cases, data from 1,133 CRC cases registered in the CRC_MSK_2017 dataset on cBioPortal (https://www.cbioportal.org/) were downloaded. We filtered 701 MSS cases (MSK_MSS_CRC_dataset) from the dataset and similarly, adjustments were made to convert these data into the file formats needed for the analyses described below.

#### Definition of prior chemotherapy exposure (number of regimens)

The number of prior systemic treatment regimens was defined according to the timing of CGP sampling. For primary tumors, regimens administered up to the date of primary tumor resection were counted. For metastatic lesions, regimens administered up to the date of metastatic lesion resection/biopsy were counted. For blood-based CGP, regimens administered up to the blood draw date were counted. No post-sampling therapies were included in the regimen count.

#### Statistical analysis of TMB and clinicopathological factors

For analysis of the relationship between TMB and various clinicopathological factors, environmental factors, and genetic variants, the Wilcoxon test was used. A p-value of < 0.05 was set as statistically significant. For plotting, the R package ggplot2 version 4.0.1 (https://cran.r-project.org/web/packages/ggplot2/index.html)^10^ was utilized.

#### OncoPrint and SNV analysis

To create an overview of oncoprint and for the SNV analysis, the R package maftools version 2.26.0 (https://bioconductor.org/packages/maftools/)^11^ was used to generate waterfall and lollipop plots. Data used included our institution’s CGP data (Supplementary Table 1 ,2, and 3) and the MSK_MSS_CRC_dataset. From each dataset, columns for Hugo_Symbol, Tumor_Sample_Barcode, Variant_Classification, Variant_Type, Chromosome, Start_Position, End_Position, Reference_Allele, Tumor_Seq_Allele2, and Protein_Position were used to create a data table. A waterfall plot was generated through oncoplot processing operations. Additionally, a lollipop plot was created using the same data.

#### Correlation analyses

For the correlation analyses, JMP version 17.0 software (SAS Institute, Inc., Cary, NC, USA; https://www.jmp.com/) was employed to conduct multivariate analysis. Data used included TMB and total number of genetic variants (missense number, amplification number, nonsense number, frameshift number, splice number, rearrangement number, loss number, and truncation number). The correlation between each variant type was analyzed. Nonparametric testing using Spearman’s rho was used for statistical tests.

#### Ethics

The ethics committee of Kurume University approved this study, which followed the principles laid down in the 1964 Declaration of Helsinki and its later amendments (approval number 445). Written informed consent was obtained from all participants.

#### Language editing and proofreading

ChatGPT (OpenAI, GPT-4 architecture, https://chatgpt.com) was used for English language editing and proofreading of the manuscript.

## Results

Over the 5 years from 2020 to 2025, CGP was done for 88 advanced CRC cases treated at our institution. An overview of these cases is presented in Table 1. The median age was 62 years, with a male to female ratio of approximately 6:4. Twenty-seven % of cases had a familial history of CRC. Synchronous cancers were observed in 11% of cases. The tissues used for CGP included primary sites in 48 cases (55%), metastatic sites in 24 cases (27%), and blood samples in 16 cases (18%). In 48 cases (32%), tissue samples from initial surgeries were used for CGP, while 63 cases (72%) had undergone chemotherapy prior to tissue samples being acquired for CGP testing. A median of 1 treatment regimen was administered. In over 82% of the cases, F1CDx (tissue based CGP) was used for CGP.

**Table 1:** Overview of the clinicopathologic and environmental factors on the enrolled patients.

Clinicopathological Factors		*N* = 88
Age	Median ± SD	62 ± 10
Sex	Male(%)/female(%)	52(59)/36(41)
Familial History of CRC	Yes(%)/no(%)/NA	23(27)/63(73)/2
Multiple Primary Cancer	Yes(%)/no(%)	10(11)/78(89)
Alcohol Habit	Never(%)/Occasional(%)/Regular(%)	54(61)/6(6.8)/28(32)
Smoking Habit	Never(%)/Past(%)/Regular(%)	44(50)/19(22)/25(28)
Tumor location	Right(%)/Left(%)	23(26)/65(74)
T	T1(%)/T2(%)/T3(%)/T4(%)/NA	3(6.1)/3(6.1)/16(33)/27(55)/39
N	N0(%)/N1(%)/N2(%)/N3(%)/NA	14(30)/11(23)/14(30)/8(17)/41
M	M0(%)/M1(%)/NA	40(55)/33(45)/15
Stage	I(%)/II(%)/III(%)/IV(%)/NA	2(2.7)/5(6.7)/31(41)/37(49)/13
Histology	Adenocarcinoma/NA	84(100)/4
Differentiation	Well(%)/mode(%)/muc(%)/NA	14(45)/16(52)/1(3.2)/57
RAS status	Wild(%)/ Mutated (%)	41(47)/47(53)
MSI status	MSS(%)/MSI(%)	88(100)/0(0)
TMB	Mean ± SD /Mb	4.2 ± 4.1
Chemotherapy Before CGP test	Yes(%)/no(%)	63(72)/25(28)
Line of Regimen	Mean ± SD	1.0 ± 1.83
CGP Test Resource	Primary Tissue(%)/Metastatic Tissue(%)/Blood(%)	48(55)/24(27)/16(18)
CGP Test Modality	F1_CDX(%)/F1L_CDX(%)	72(82)/16(18)

SD: standard deviation, T1: , T2: ,T3:, T4:, N0, N1, N2, N3, M0:, M1:, well: well differentiated, mode: moderately differentiated, muc: mucinous, MSS: microsatellite stable, MSI: microsatellite instable, TMB: tumor mutation burden, CGP: comprehensive genome profiling, F1_CDX: FoundationOne CDX, F1L_CDX: FoundationOne Liquid CDX, NA: not available.

The regimen count was anchored to the CGP sampling time point for each specimen (resection/biopsy date for tissue, blood draw date for liquid biopsy), and post-sampling treatments were not included.

### Analysis of association with TMB

The median ± standard deviation TMB value among the 88 cases was 4.2 ± 4.1. To identify the type of genetic variant most strongly associated with TMB, a correlation analysis was conducted. As shown in Supplementary Fig. 1, a strong positive correlation was observed with the total variant number, particularly with SNVs, including missense variant numbers. Given this association, subsequent analyses focused on the association between TMB, SNVs, and clinicopathological factors.

#### Impact of prior chemotherapy exposure on TMB

**Fig. 1:**
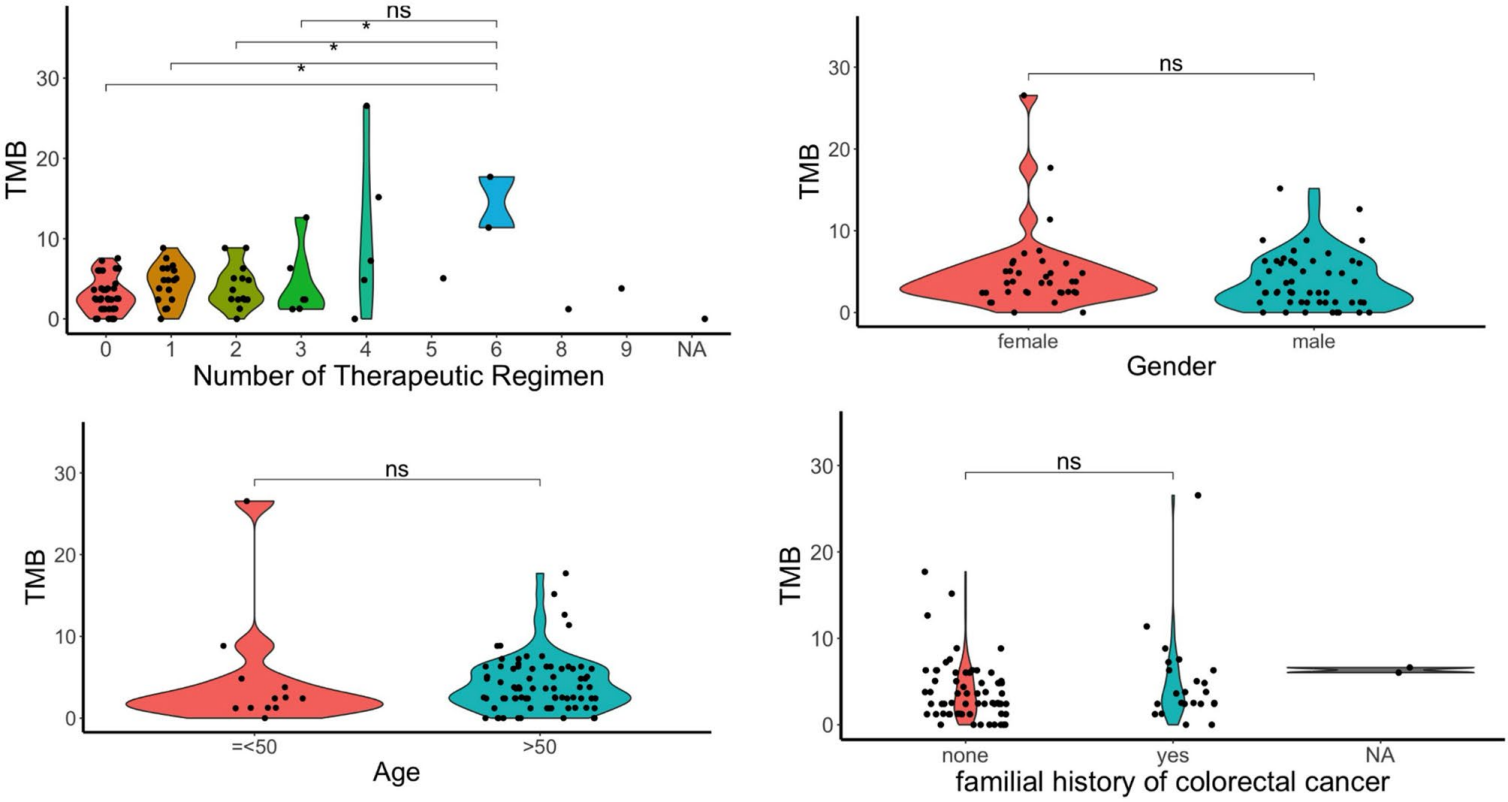
Statistical analysis of TMB and various clinicopathological factors. Student’s t-test and Wilcoxon test were used for statistical analysis. *P* value < 0.05 was defined as statistical significance. Top left shows the relationship between chemotherapy regimen number and TMB. The x-axis indicates the number of treatment regimens. The top right shows the relationship between gender and TMB. The bottom left shows the relationship between age and TMB. The bottom right panel shows the relationship between having a family history of colorectal cancer and TMB. NA: not available, ns ; no significant difference, *: statistical significance.

To clarify the effect of chemotherapy exposure on TMB we examined the relationship between the number of chemotherapy regimens administered and TMB. As shown in Fig. 1 (upper left), the higher regimen numbers were statistically associated with higher TMB . All analyzed cases were microsatellite stable (MSS). Variants in *POLE* or *POLD1* were detected in four cases each; however, all variants were located outside the exonuclease domain or were classified as variants of uncertain significance, and none were associated with elevated TMB (Supplementary Fig. 2 and 3). These findings suggest that the observed variation in TMB was unlikely to be driven by intrinsic polymerase-related mechanisms.

We further analyzed the impact of the specific chemotherapeutic agents to which each case had been exposed on TMB. However, no statistically significant association was observed between TMB values and differences in drug class or mechanism of action in this analysis.(Supplementary Fig. 4).

#### Association between TMB and clinicopathological factors

**Fig. 2:**
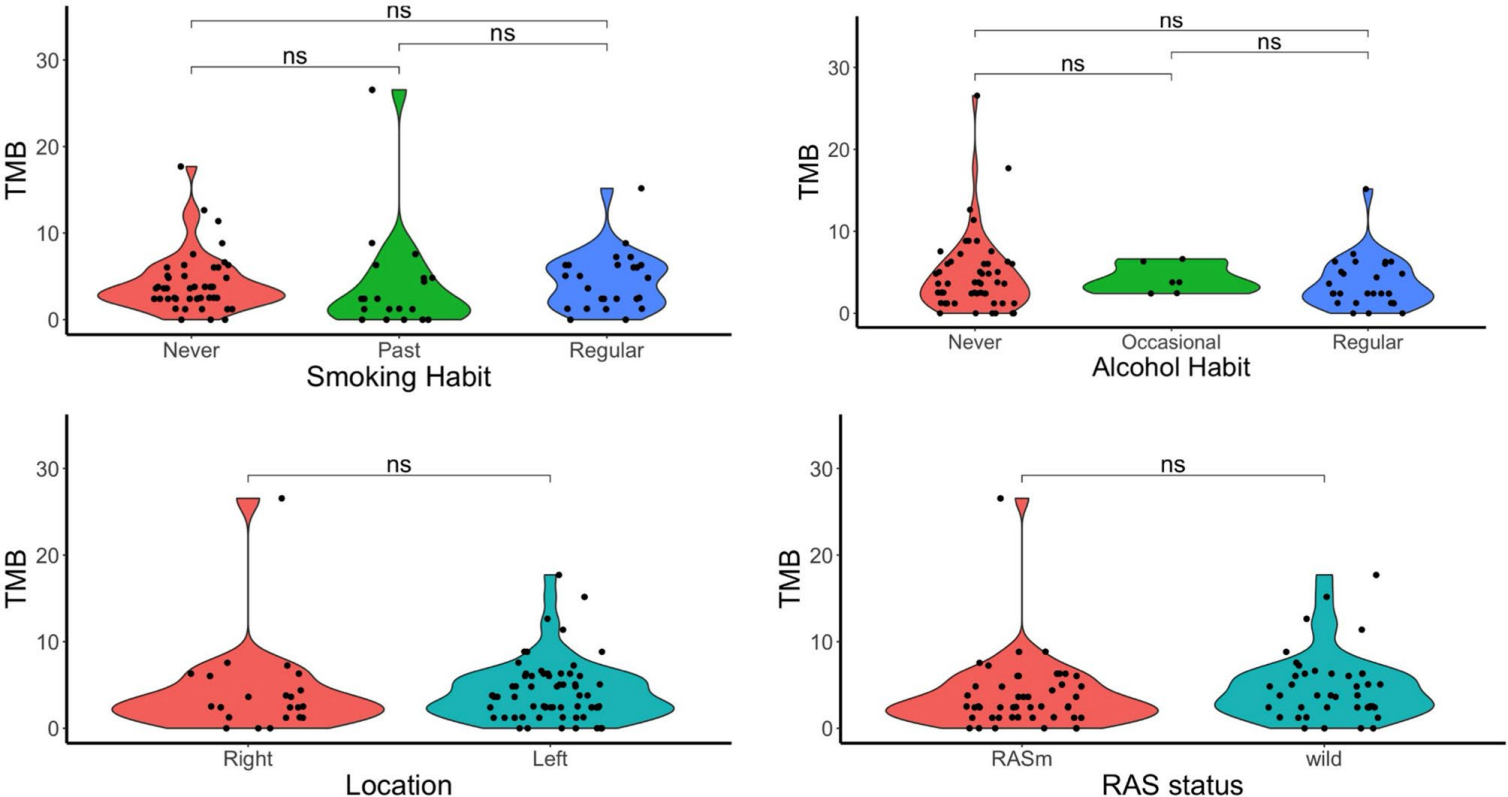
A statistical analysis was conducted using the Wilcoxon test. The Y-axis represents TMB in all plots. Top left: The relationship between smoking history and TMB. Top right: The relationship between alcohol consumption and TMB, categorized as Never (no history of drinking), Occasional (social drinking), and Regular (routine drinking). Bottom left: The relationship between primary tumor location and TMB, categorized as Right (from ascending to transverse colon), and Left (from descending to rectum). A p-value of < 0.05 was considered statistically significant. Ns; no significant difference.

Subsequently, statistical analyses of clinical, pathological, and environmental elements were conducted to elucidate any associations with TMB. As shown in Fig. 1 (upper right and bottom), no statistical differences were observed between TMB and sex, age, familial history of colorectal cancer. In addition, no statistically significant associations were observed between tumor mutation burden and smoking history, alcohol history, primary tumor location, or RAS mutation status (Fig. 2).

#### Overview of a genetic variants profile

Furthermore, to characterize the genomic variants profile of CRC cases treated at our institute, tumor genomic variants analyses were performed. A total of 1,138 single nucleotide variants (SNVs) were identified, with missense mutations being the most frequent (861 variants, 76%), followed by nonsense mutations (109 variants, 9.6%), while the remaining variants consisted of frameshift insertions and deletions, splice-site variants, and in-frame insertions or deletions. In addition, 339 copy number variants (CNVs) were detected, predominantly amplifications (323 events, 95%), with losses observed in 16 cases (5%). Structural variants comprised 32 events, mainly rearrangements (59.4%) and truncations (25%), and three gene fusions (9.4%) were identified, along with one duplication and one deletion (Table 2).

**Table 2:** Variant Overview.

SNVs	N = 1,138 (%)
Missense_Mutation	861 (76)
Nonsense_Mutation	109 (9.6)
Frame_Shift_Deletion	64 (5.6)
Frame_Shift_Insertion	41 (3.6)
Splice_Site	27 (2.4)
In_Frame_Deletion	19 (1.7)
In_Frame_Insertion	17 (1.5)

CNVs	N = 339 (%)
Amplification	323 (95)
loss	16 (5)

Structure Variants	N = 32 (%)
Rearrangement	19 (59.4)
Truncation	8 (25)
Fusion	3 (9.4)
Duplication	1 (3.1)
Deletion	1 (3.1)

SNVs: Single Nucleotide Variants, CNVs: Copy Number Variants

**Fig. 3:**
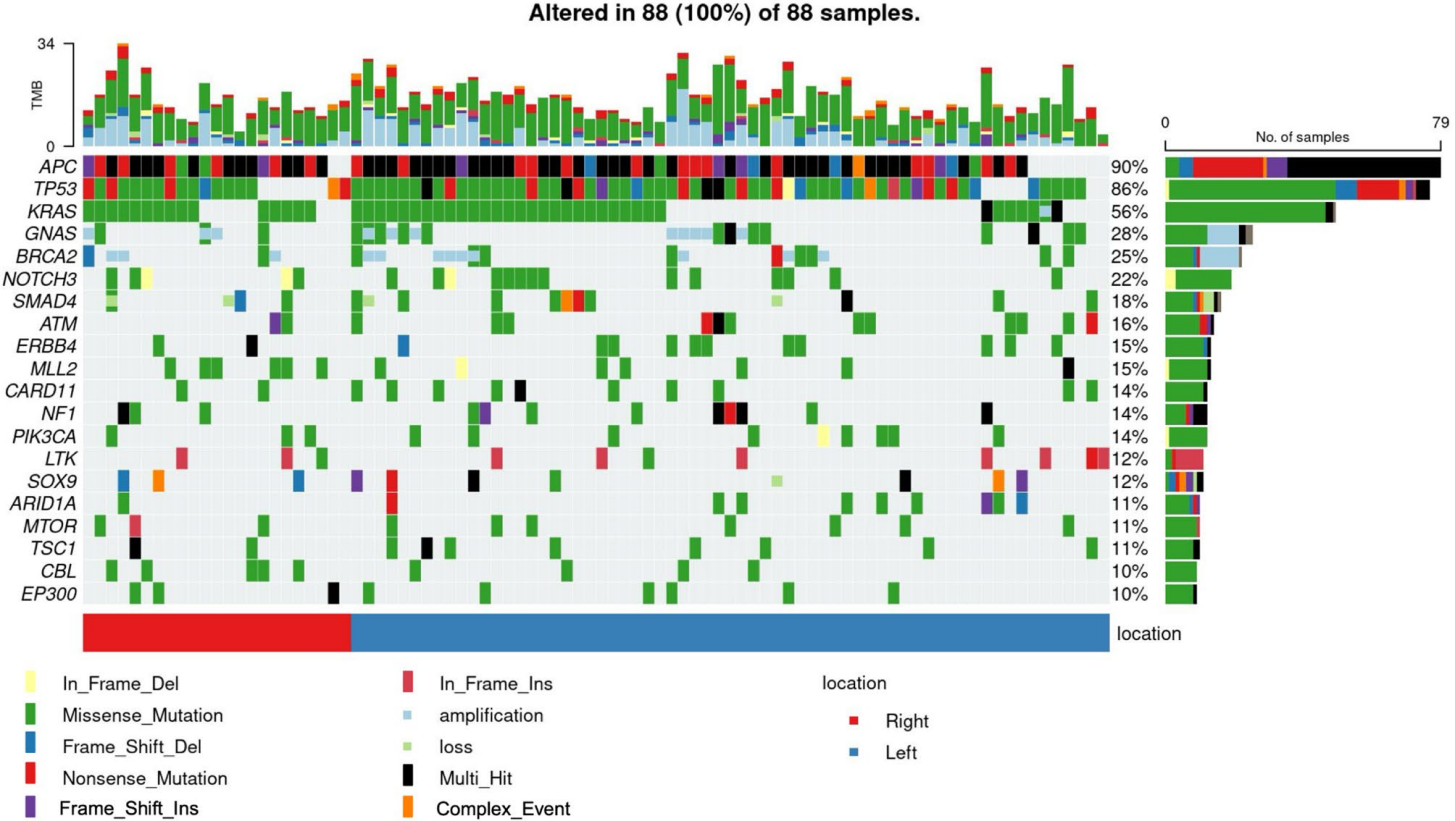
Figure showing the top 20 genetic variants observed in the registered cases (*n* = 88). All cases were aligned along the X-axis and each genetic variant was plotted along the Y-axis. The left vertical axis displays the names of the genes. The numbers on the right-hand vertical axis indicate the total percentage of each gene alteration. The bar chart on the right shows the total number and composition of each gene variant. The bar plot at the top indicates the number of gene alterations observed per case. The lower chart shows the primary location of the tumour. Right: From the ascending colon to the transverse colon. Left: from the descending colon to the rectum. The bottom of the legend uses colour-coded boxes to represent each type of genomic variant. The legend includes Nonsense_Mutation: nonsense variants, In_Frame_Del:in-frameshift deletions, Missense_Mutation: missense variants, Frame_shift_Del: frameshift deletions, Splice_Site: splice site variants, Frame_Shift_Ins: frameshift insertion variants, In_Frame_Ins: in-frameshift insertion variants, and Multi_Hit: multi-hit variants.

Figure 3 presents an overview of the genetic variants revealed following CGP. Among the top 20 genetic variants, the three most common were *APC* variants (90% of cases), *TP53* variants (86% of cases), and *KRAS* variants (56% of cases). In decreasing order of frequency, additional variants were identified in *GNAS*, *BRCA2*, *NOTCH3*, *SMAD4*, *ATM*, and *ERBB4*. Approximately half of the *BRCA2* and *GNAS* variants were amplifications or single nucleotide variants (SNVs), while the majority of the other genetic variants were SNVs.

#### Comparison of SNVs in the present study with public data

**Fig. 4:**
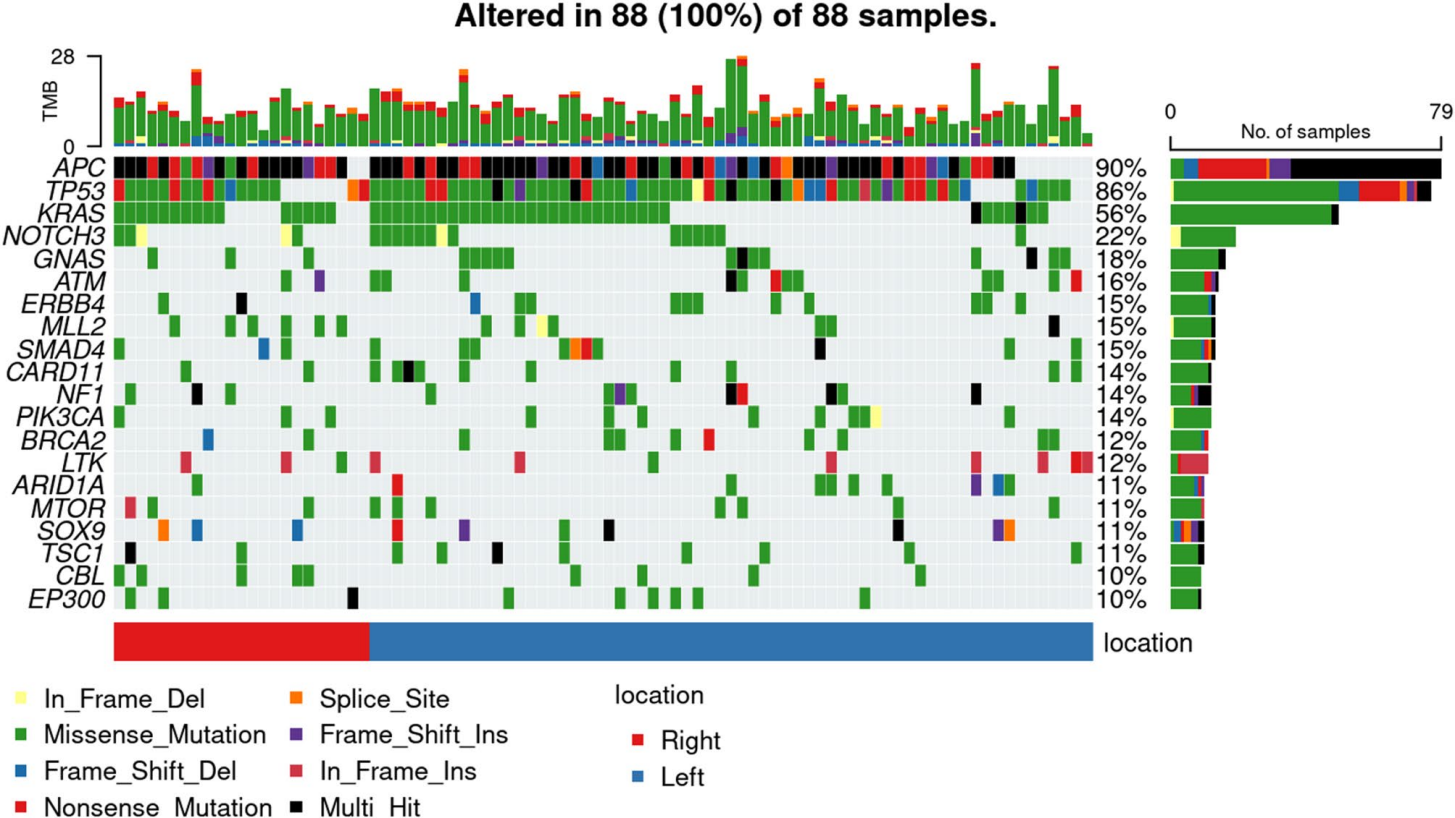
Top twenty of single nucleotide variants data from CGP performed at our facility (*N* = 88). The left vertical axis plots the genes in which variants were confirmed, arranged in order of frequency. The right vertical axis shows the frequency with which variants in the corresponding genes were observed across all cases. The bar plot on the right displays the number of cases in which variants were detected. The upper part of the plot shows the total number of variants per case. The lower chart shows the primary location of the tumour. Right: From the ascending colon to the transverse colon. Left: from the descending colon to the rectum. The bottom legend part of the plot illustrates the types of variants, each indicated by different colors. The legend includes Nonsense_Mutation: nonsense variants, In_Frame_Del:in-frameshift deletions, Missense_Mutation: missense variants, Frame_shift_Del: frameshift deletions, Splice_Site: splice site variants, Frame_Shift_Ins: frameshift insertion variants, In_Frame_Ins: in-frameshift insertion variants, and Multi_Hit: multi-hit variants.

Figure 4 displays the genes in which SNVs were observed. Variants in *APC* and *TP53* were found in 90% and 86% of cases, respectively, and *KRAS* variants were identified in 56% of cases. When compared with the MSK_MSS_CRC_dataset (Supplementary Fig. 5), the three most common genes remained consistent; however, the frequency of variants in these genes in the present study dataset were approximately 10 to 20% higher. Additionally, the next most common genes having variants in the MSK_MSS_CRC_dataset were *PIK3CA*, *SMAD4*, *BRAF*, and *FBXW7*, whereas in the present study dataset, the next most common genes having variants were *NOTCH3*, *GNAS*, *ATM*, and *ERBB4*.

**Fig. 5:**
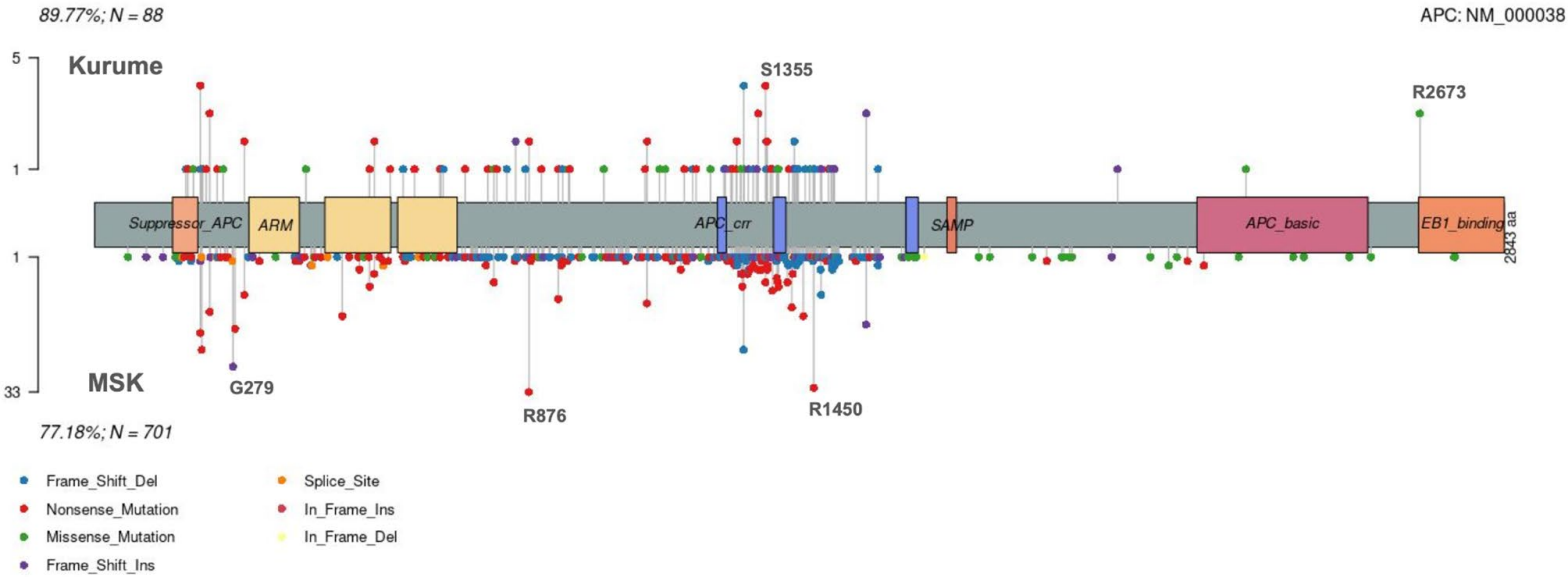
The upper part of the lollipop plot displays *APC* variants identified in the present study, while the lower part shows the variants observed in public data. Each bar in the plots represents the position of the variant on the gene, and the left Y-axis indicates the number of cases in which the variants were confirmed. The X-axis indicates the position of the gene. The Y-axis shows the number of cases exhibiting the variant in each dataset. The box plot at the top of the X-axis illustrates the domains of the gene. Domain Name, Suppressor_*APC*: Suppressor domain of *APC*, *ARM*: Armadillo repeats, *APC_crr*: *APC* catenin regulatory region, *SAMP*: Serine-Alanine-Methionine-Proline repeat, *APC*_basic: Basic domain of *APC*, *EB1*_binding: End-binding protein 1 binding domain.

**Fig. 6:**
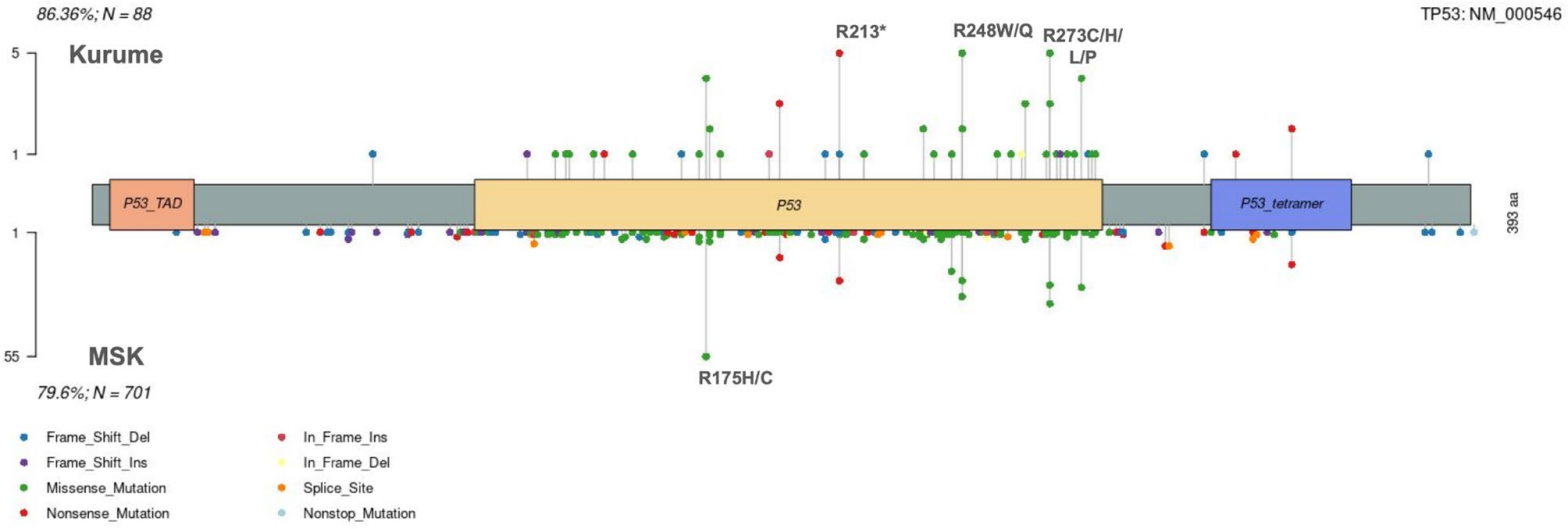
The upper section of the lollipop plot illustrates *TP53* variants identified in the present study, while the lower section shows variants found in public data. Each bar in the plots represents the location of the variant on the gene, and the Y-axis indicates the number of cases in which the variants were confirmed in each dataset. The box plot at the top of the X-axis depicts the domains of the gene. Domain Name, *TP53_TAD* : TP53 transactivation domain, *P53_tetramer* : p53 tetramerization domain.

**Fig. 7:**
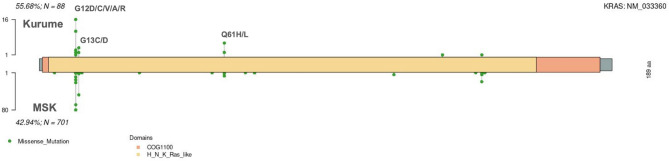
The upper section of the lollipop plot displays *KRAS* variants identified in the present study, while the lower section shows variants found in public data. Each bar in the plots indicates the position of the variant on the gene, and the Y-axis denotes the number of cases in which these variants were confirmed in each dataset. The box plot at the top of the X-axis illustrates the domains of the gene. Domain Name: COG1100: Small GPT-binding *RAS* family domain, H_N_K_Ras_like: Ras-like small GTPase nucleotide-binding domain.

A comparison of the variants observed among the three most common genes between the present study dataset and the MSK_MSS_CRC_dataset was also conducted. As shown in Fig. 5, *APC* variants in the present study showed peaks at p.1355 and p.R2673, whereas no prominent peaks at these positions were identified in the MSK_MSS_CRC_dataset. Additionally, the peaks observed at p.G279, p.R876, and p.R1450 in the CRC_2017_MSK dataset were not seen in the present study dataset. As depicted in Fig. 6, the overall profile of *TP53* variants appears similar between datasets, although the frequency of variants at p.R213*, p.R248W/Q, and p.G273 was relatively higher in the present study dataset compared with the MSK_MSS_CRC_dataset. Regarding *KRAS*, as shown in Fig. 7, no significant differences in variant frequency other than p.Q61 were noted between the present study and MSK_MSS_CRC_dataset.

**Fig. 8:**
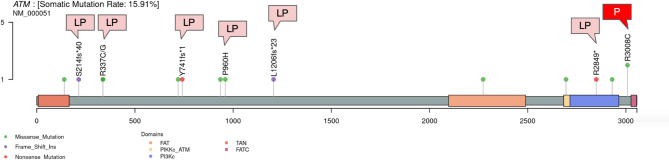
The lollipop plot above shows genetic variants in the *ATM* gene identified by this analysis. Each bar plot indicates the variant position. Variants labeled above their position are assessed as likely pathogenic or pathogenic. The flag symbols above the labels LP: likely pathogenic, P : pathogenic. Unlabeled positions are variants of unknown significance (VUS). The X-axis of the box plots indicates the gene domain. The Y-axis shows the number of cases exhibiting the variant. Domain names: FAT:FRAP-ATM-TRRAP domain, PIKKc_ATM: Phosphatidylinositol 3-kinase-related kinase catalytic domain, PI3Kc: Phosphatidylinositol 3-kinase catalytic domain, TAN: Tel1/ATM N-terminal domain, FATC: FRAP-ATM-TRRAP C-terminal domain.

Among the genetic variants ranked below the top three, *ATM* variants were observed at a relatively higher frequency in the present dataset compared with the MSK_MSS_CRC_datase (15.91% vs 5.14%, Fig. 4 and Supplementary Fig. 5). In the present cohort, *ATM* variants were identified in a total of 15 cases, of which more than half (9 cases) were classified as likely pathogenic or pathogenic (Fig. 8).

## Discussion

In this study, a significant association was identified between the number of treatment regimens and TMB. Cytotoxic chemotherapy is known to induce DNA damage through mechanisms such as crosslink formation and strand breaks^12,13^. In CRC, therapeutic history was reported to affect TMB and genetic alteration spectrum with supporting our finding^14^.

The presence of *POLE/POLD* gene variants in CRC has been reported to be associated with the development of an ultra-hypermutated phenotype and improved responses to immune checkpoint inhibitor therapy^5,9,15^. In this study, we analyzed the correlation between the presence of variants in *POLE/POLD1* genes and TMB; however, all detected variants were classified as non-pathogenic. Accordingly, MSS TMB-high CRC cases identified in this cohort were not driven by mutations in *POLE* or *POLD1* genes. Neither alcohol nor smoking history was associated with TMB in this cohort. In the absence of pathogenic *POLE/POLD1* alterations, these findings suggest that elevated TMB in MSS CRC may reflect cumulative influences of treatment history and tumor evolutionary processes rather than intrinsic hypermutagenic processes.

On the other hand, studies have reported that DNA damage-type therapies are involved in changes in genetic mutation profiling^16,17^, thus we attempted a correlative analysis between TMB level and chemotherapeutic agents. But, there were no clear differences in TMB that were observed according to the class of chemotherapeutic agents used. Further investigation into the duration and intensity of chemotherapy exposure, along with enhanced pharmacodynamic effect information based on chemotherapy combinations and increased case numbers, may yield similar relationships. However, combining the two results above indicates that while the relationship between pre-CGP chemotherapy exposure and TMB is associative at this time, it has not yet been established as causal.

Such acquired mutations may not necessarily confer the immunogenicity typically associated with polymerase-driven hypermutation, and therefore may not translate into sensitivity to immune checkpoint inhibitors. These observations align with recent reports indicating that MSS tumors with high TMB represent biologically diverse entities and do not consistently exhibit sensitivity to immune checkpoint blockade^18^.

The three most common genes in which SNVs were confirmed in the present study (*APC*, *TP53*, and *KRAS*) matched those in the public dataset. However, the next most common genes with variants differed between the present study and public datasets. While these genes participate in common pathways related to CRC development, the genetic alterations contributing later-stage tumor characteristics may differ between cases treated at our institution and those in public databases. Such differences may reflect multiple factors, including patient background and sampling context of this cohort.

The differences in genetic variants observed beyond the top three in the present analysis are likely to reflect differences in cohort characteristics, disease stage, prior treatment exposure, and sample selection between the two datasets. Among them, the *ATM* gene is involved in the DNA damage checkpoint response pathway involved in maintaining genomic stability^19,20^, In the present cohort, ATM variants were observed at relatively higher frequency; however, due to the limited sample size, their potential relationship with TMB or treatment exposure could not be determined. Further studies in larger cohorts are required to clarify their clinical and biological significance.

### Limitations

A key limitation of this study is the small sample size (*n* = 88), which limits the generalizability of findings regarding characteristic SNV profiles and the observed association between higher TMB and greater numbers of prior treatment regimens. In addition, follow-up data for TMB-high patients treated with ICIs were not available, precluding assessment of the clinical relevance of elevated TMB.

Changes in TMB may reflect not only exposure to chemotherapy but also genetic alterations within the tumor itself over time. As noted in the methods section, to assess solely the impact of chemotherapy, the number of treatment cycles performed up to the time of sample submission for CGP testing is counted, and any subsequent cycles are omitted. However, depending on the storage conditions and age of the analyzed samples, DNA degradation may occur, potentially leading to the detection of such changes as genetic variants.

This analysis was restricted to SNVs and lacked quantitative data on smoking and alcohol exposure, limiting interpretation of potential environmental influences. Selection bias may also be present, as CGP was performed in clinically selected advanced cases. Furthermore, differences between tissue-based and blood-based assays introduce the possibility that some detected variants may originate from clonal hematopoiesis rather than tumor tissue, which could influence mutational estimates.

Finally, variability in CGP sampling timing and incomplete time-to-sampling data prevented adjustment for disease duration and temporal confounding. Therefore, the association between treatment exposure and TMB should be interpreted as associative and hypothesis-generating rather than causal.

## Conclusion

In this study, comprehensive genomic profiling of advanced colorectal cancer revealed an association between the number of prior chemotherapy regimens and tumor mutation burden. MSS TMB-high cases were not driven by polymerase gene alterations, suggesting that treatment exposure may be associated with mutational accumulation. These findings indicate that TMB should be interpreted in the context of therapeutic history, as elevated TMB may not necessarily reflect intrinsic tumor biology or predict sensitivity to immune checkpoint inhibitors.

## Supplementary Information

Below is the link to the electronic supplementary material.

Supplementary Material 1

Supplementary Material 2

Supplementary Material 3

Supplementary Material 4

## Data Availability

The supplementary data (Supplementary Table 1, Table 2, and Table 3) generated in the present study are available in the following data repository. 10.6084/m9.figshare.30081775.

## Abbreviations

TMB: Tumor mutation burden
CRC: Colorectal cancer
CGP: Comprehensive genomic profiling
XML: Extensible markup language
MSK: Memorial sloan kettering
SNV: Single nucleotide variant
MSI: Microsatellite instability
dMMR: Mismatch repair deficiency
ICIs: Immune checkpoint inhibitors
MSS: Microsatellite stable
F1CDx: FoundationOne CDx
F1LCDx: FoundationOne Liquid CDx
EP: Expert panel
VAF: Variant allele fraction
WBC: White blood cell
