# Supplementary material for: Impact of chemotherapy exposure on tumor mutation burden in advanced colorectal cancer
Source: Sci Rep. 2026 Apr 6;16:16380. doi: 10.1038/s41598-026-46050-7 (PMC13212886; doi:10.1038/s41598-026-46050-7)
Supplement: Supplementary file 4 — Supplementary Material 4 [file 41598_2026_46050_MOESM4_ESM.pdf]

Supplementary Figure 1

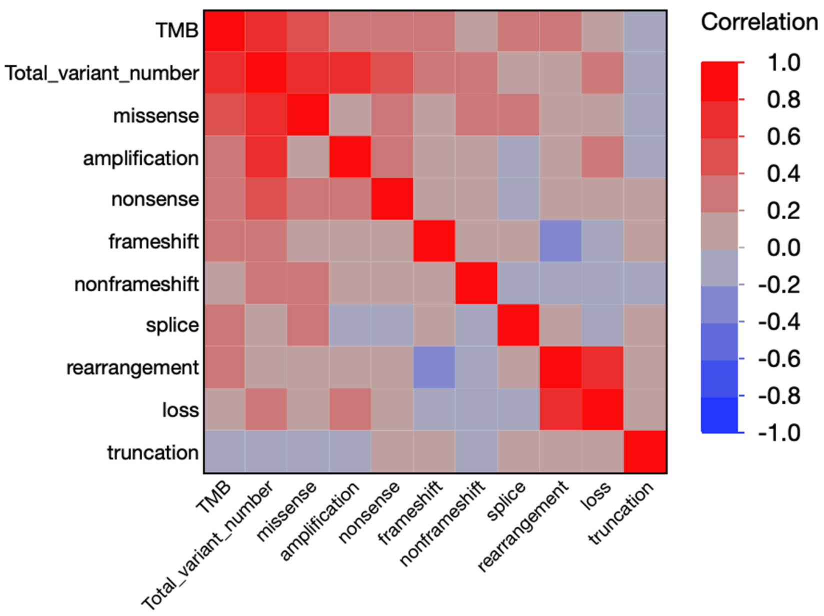

The correlations among different variant types were analyzed using correlation analysis. The correlation plot on the right displays the degree of correlation, with red indicating a positive correlation and blue indicating a negative correlation. The X and Y-axes indicate the type of genetic variant.

Supplementary Figure 2

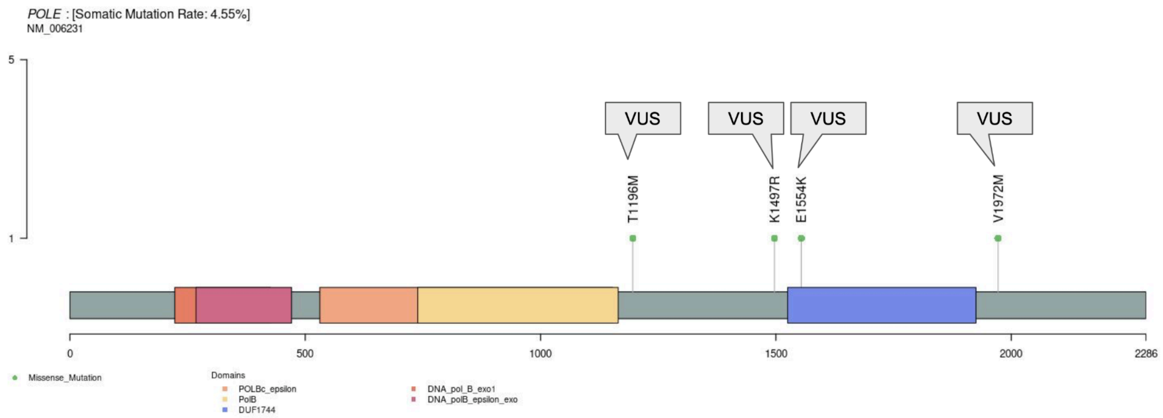

The lollipop plot above shows genetic variants in the *POLE* gene identified by this analysis. Each bar plot indicates the variant position. The flag symbols above the labels VUS: variants of unknown significance (VUS). The X-axis of the box plots indicates the gene domain. The Y-axis shows the number of cases exhibiting the variant. Domain names: POLBc_epsilon: DNA polymerase B catalytic subunit epsilon, PolB: DNA polymerase family B, DUF1744: Domain of unknown function 1744, DNA_pol_B_exo1:DNA polymerase family B exonuclease domain 1, DNA_polB_epsilon_exo: DNA polymerase epsilon proofreading exonuclease domain.

Supplementary Figure 3

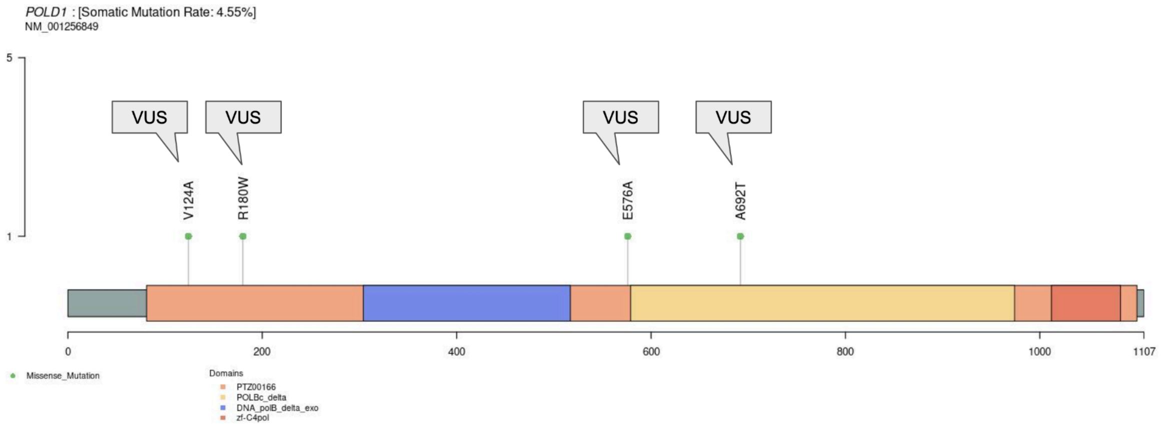

The lollipop plot above shows genetic variants in the *POLD1* gene identified by this analysis. Each bar plot indicates the variant position. The flag symbols above the labels VUS: variants of unknown significance (VUS). The X-axis of the box plots indicates the gene domain. The Y-axis shows the number of cases exhibiting the variant. Domain names: PT1200166: polymerase delta regulatory-associated domain, POLBc_delta: DNA polymerase B catalytic subunit delta , DNA_polB_delta_exo: DNA polymerase delta proofreading exonuclease domain, zf-C4pol: Zinc-finger C4-type polymerase domain.

Supplementary Figure 4

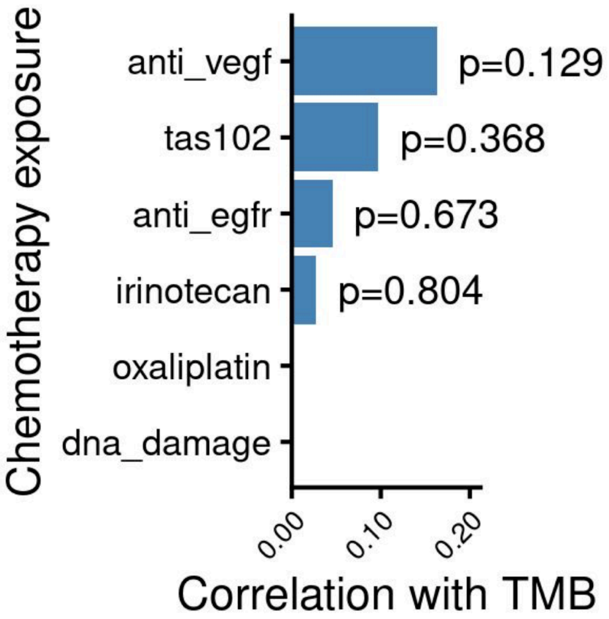

Statistical analysis of the relationship between tumour mutation burden (TMB) and each chemotherapeutic agent. X-axis: TMB correlation coefficient.Y-axis: each chemotherapeutic agent. Anti-VEGF: anti-vascular endothelial growth factor agent. Tas102: trifluridine/tipiracil. Anti-EGFR: anti-epidermal growth factor receptor agent. DNA_damage: total exposure to DNA damage agents. A p-value of <0.05 was defined as statistically significant.

Supplementary Figure 5

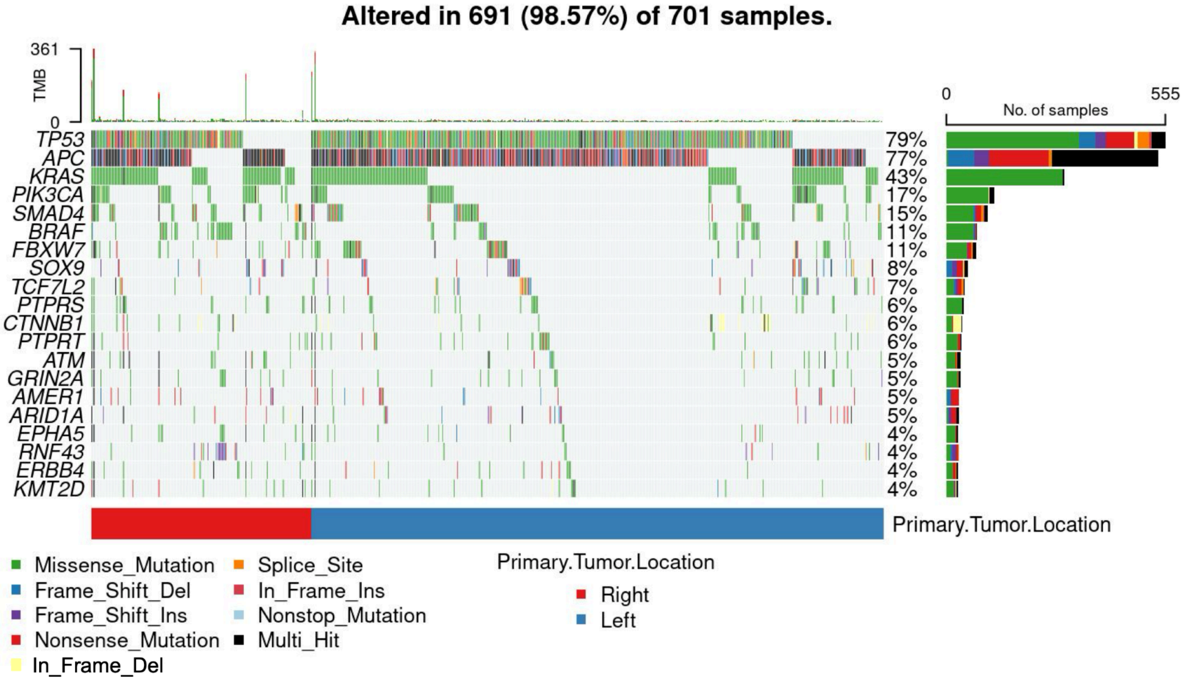

Top twenty of single nucleotide variant data from MSK_MSS_CRC_datase (N=701). The left vertical axis plots the genes in which variants were confirmed, arranged in order of frequency. The right vertical axis shows the frequency with which variants in the corresponding genes were observed across all cases. The bar plot on the right displays the number of cases in which variants were detected. The upper part of the plot shows the total number of variants per case. The lower chart shows the primary location of the tumour. Right: From the ascending colon to the transverse colon. Left: from the descending colon to the rectum. The bottom legend part of the plot illustrates the types of variants, each indicated by different colors. The legend includes Nonsense_Mutation: nonsense variants, In_Frame_Del:in-frameshift deletions, Missense_Mutation: missense variants, Frame_shift_Del: frameshift deletions, Splice_Site: splice site variants, Frame_Shift_Ins: frameshift insertion variants, In_Frame_Ins: in-frameshift insertion variants, and Multi_Hit: multi-hit variants.
